# The Molecular Photo-Cell: Quantum Transport and Energy Conversion at Strong Non-Equilibrium

**DOI:** 10.1038/srep08312

**Published:** 2015-02-09

**Authors:** Shigeru Ajisaka, Bojan Žunkovič, Yonatan Dubi

**Affiliations:** 1 Department of Chemistry, Ben-Gurion University of the Negev, Beer-Sheva 84105, Israel; 2 Departamento de Física, Facultad de Ciencias Físicas y Matemáticas, Universidad de Chile, Casilla 487-3, Santiago Chile; 3 Ilse-Katz Institute for Nanoscale Science and Technology, Ben-Gurion University of the Negev, Beer-Sheva 84105, Israel

## Abstract

The molecular photo-cell is a single molecular donor-acceptor complex attached to electrodes and subject to external illumination. Besides the obvious relevance to molecular photo-voltaics, the molecular photo-cell is of interest being a paradigmatic example for a system that inherently operates in out-of-equilibrium conditions and typically far from the linear response regime. Moreover, this system includes electrons, phonons and photons, and environments which induce coherent and incoherent processes, making it a challenging system to address theoretically. Here, using an open quantum systems approach, we analyze the non-equilibrium transport properties and energy conversion performance of a molecular photo-cell, including both coherent and incoherent processes and treating electrons, photons, and phonons on an equal footing. We find that both the non-equilibrium conditions and decoherence play a crucial role in determining the performance of the photovoltaic conversion and the optimal energy configuration of the molecular system.

Understanding the properties of non-equilibrium systems has been a central effort of the scientific community for many years. Of specific interest are non-equilibrium processes that take place at the nano-meter scale and at which energy is converted from one form to another, for instance photovoltaic (PV) energy conversion, photochemistry and photosynthesis. In these cases, the interaction between electrons and photons under non-equilibrium conditions plays an essential role. Theoretical modeling of such processes is a challenging task, since the interacting nature of the system and its many-body characteristics, the multitude of constituents, the presence of external environments, and the non-equilibrium conditions must all be taken into consideration.

Even harder to address theoretically are situations in which the system has two independent fluxes, originating from separate non-equilibrium drivings, and are both far away from the linear response regime, a situation which is designated as *strong non-equilibrium*. A paradigmatic example for such a system is photo-voltaic cells, where the two fluxes are the heat flux, originating from the huge temperature difference between the sun and the earth, and the particle current originating from the bias voltage between the electrodes. In recent years a new and exciting class of photo-voltaic cells has emerged, namely molecular photo-cells, where the energy conversion process takes place at the single molecule level^1^,^2^,^3^.

Here we propose a formalism to study non-equilibrium transport in molecular junctions, and use it to investigate a model for the molecular photo-cell, a single molecular donor-acceptor complex attached to electrodes and subject to external illumination. This model was recently suggested^4^,^5^,^6^ (and a simpler version in Ref. 7) to be the minimal model to describe PV energy conversion in ideal, single-molecule heterojunction organic PV cells. In Refs. 4, 5 PV conversion efficiency was analyzed using the (essentially classical) rate equations for the electronic degrees of freedom. The dynamics and non-equilibrium properties of the phonons and photons were ignored, being considered only within a (non-self-consistent) mean-field approximation and assumed to have equilibrium distributions. Here we show that the non-equilibrium properties of the phonons and photons have a strong impact on the PV conversion properties in realistic parameter range and cannot be neglected.

The formalism we present here allows us to treat electrons, photons and phonons fully quantum mechanically and on an equal footing (without resorting to a mean-field approximation) and to take into account the action of the environments producing a strong non-equilibrium situation. We use the many-body Lindblad quantum master equation^8^,^9^,^10^ to describe the environments, which consist of metallic electrodes in touch with a molecular complex, a phonon bath (at ambient temperature) and a photon bath (at the solar temperature). Non-equilibrium is induced by two sources, namely the temperature difference between the incoming photons (originating from the sun) and the phonons with ambient temperature, and the bias voltage between the electrodes.

Using this formalism, we calculate the non-equilibrium densities of electrons, photons and phonons, the electric current and power output, and the thermodynamic efficiency at maximal power. We find that under certain conditions, the distribution functions for the phonons and photons can be very different from the equilibrium distributions, and therefore approximating the system as close to equilibrium is not a valid approximation. We then study the signature of non-equilibrium on the energy conversion efficiency of the system.

In addition to be able to include strong non-equilibrium effects and to account for all constituents on equal footing, our formalism allows one to introduce effects of dephasing and decoherence in a simple and physically transparent way. We study how the loss of quantum coherence affects the efficiency, and show that classical electron transfer (as opposed to coherent electron propagation) *enhances* the efficiency.

## Results: efficiency for the coherent system far from equilibrium

**Figure 1:**
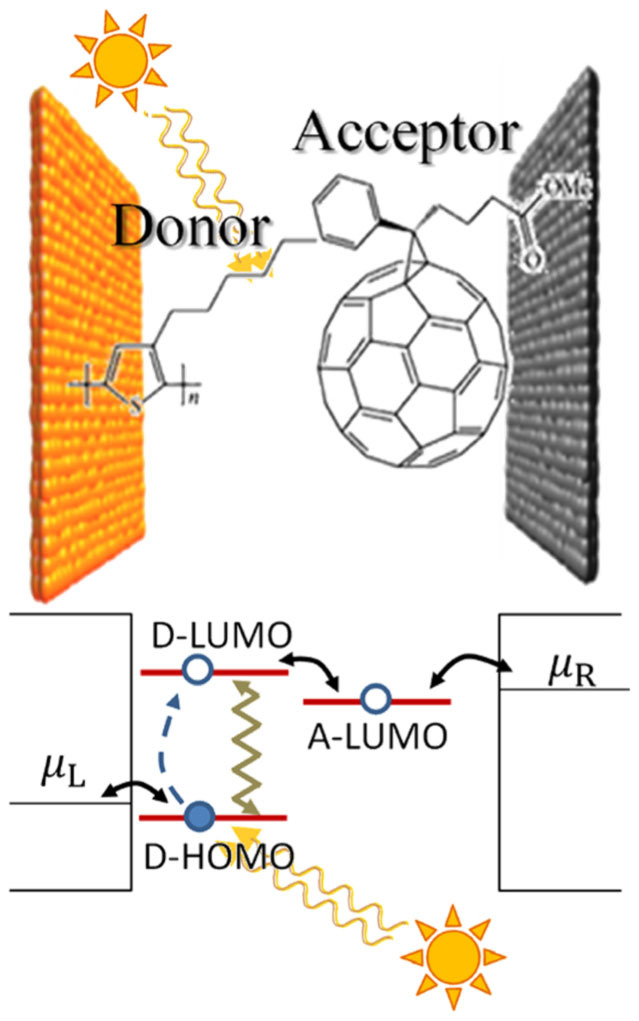
Schematic illustration of the minimal model for a molecular PV cell. The system consists of a molecule donor and an acceptor molecule, characterized by their HOMO and LUMO levels and coupled to each other via electron hopping. The D-molecule is coupled only to the left electrode, and the A-molecule only to the right electrode. Electrons in the donor interact with both photons (wiggly line) and phonons (broken line).

The system under consideration is composed of a molecular junction, in which a donor-acceptor (D-A) complex is placed between two metallic electrodes (Fig. 1)^4^. This is an idealization of an envisioned future single-layer molecular photovoltaic cells, where a self-assembled layer of D-A pairs is placed on a conducting substrate, and is covered by a top transparent electrode. For the donor (D), we consider the highest occupied molecular orbital (HOMO) with energy 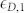 and lowest unoccupied molecular orbital (LUMO) with energy 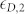. For the acceptor (A), we consider only the LUMO with energy 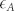 (the energy of the A's HOMO is much lower and is unaffected by any dynamics in the photocell^4^). The reader is referred to the Methods section and supplementary materials for a detailed description of the model and calculation. Recent advances in the experimental ability to measure photo-conductivity and PV conversion in single-molecule junctions^11^,^12^,^13^,^14^,^15^ make our theoretical model experimentally relevant.

**Figure 2:**
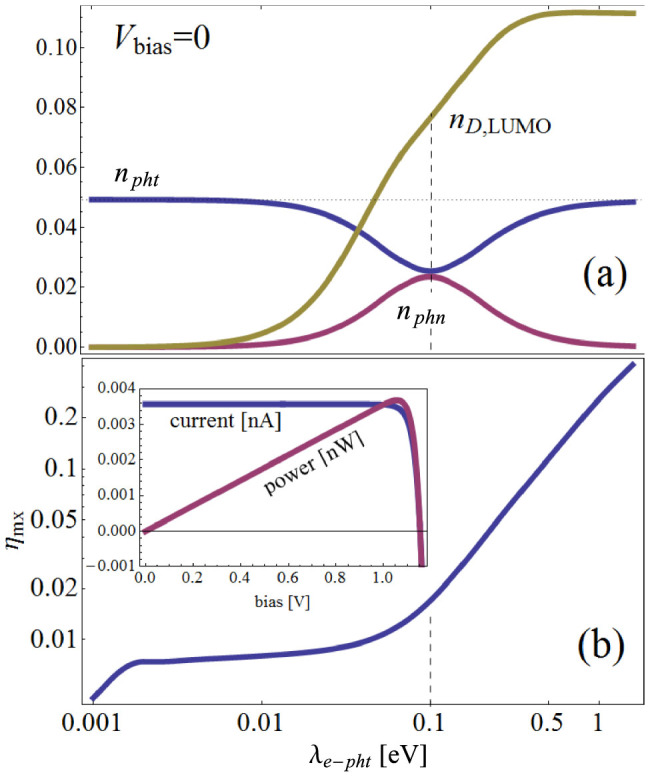
(a) Photon density *n_pht_*, phonon density *n_phn_* and the electronic occupation of the donor LUMO as a function of *e – pht* coupling *λ_epht_*. Dashed line indicates the value of *λ_ephn_*. Dotted horizontal line marks the equilibrium occupation of solar photons. (b) Efficiency at maximum power *η*_mx_ as a function of the *e – pht* coupling (same parameters as in (a)). Inset: typical current *J* and output power *P*_out_ = *J* × *V* vs. bias voltage *V*.

We begin by examining the electron, photon and phonon densities at zero bias voltage. Note that the system is still out of equilibrium due to the temperature difference between the photon and phonon baths. In Fig. 2(a) steady state averages of the photon density 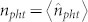, the phonon density 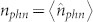, and the electronic occupation of the D-LUMO, 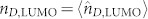, are plotted as a function of *e*–*pht* coupling *λ_e_*_–*pht*_, where 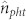, 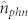 and 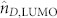 are particle number operators of the photon, the phonon, and the D,LUMO, respectively. Here, 〈·〉 represents the steady state average, i.e., 〈·〉 ≡ tr(·*ρ*_S*S*_) with the steady state density operator *ρ*_S*S*_ (see supplementary material). We set the orbital energies to be 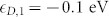, 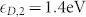, and 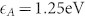
4. The *e – phn* interaction is fixed at *λ_e–phn_* = 0.1 eV (dashed vertical line in Fig. 2(a)). Three distinct regimes are observed: (i) At small *e – pht* coupling, 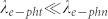, the system is close to equilibrium, and the photon occupation is defined by the solar temperature *T_s_* (dotted line). The phonons are excited according to the ambient temperature (which is very small compared to *ω*_0_, and consequently the phonon occupation is very small), and the occupation of the D-LUMO level is also very small. (ii) As the *e – pht* and *e – phn* interactions become comparable, energy is transferred from the photons to the phonons, mediated by excitation of electrons from the D-HOMO to the D-LUMO level. As a result, the D-LUMO and phonon occupations increase, while the photon occupation decreases. Alternatively, this situation can be described in terms of heating (although the notion of temperature is not applicable out of equilibrium, it is still useful to think in terms of an effective temperature): the junction is locally (and efficiently) heated by the photons. This heat is transferred to the phonons, resulting in an elevated effective phonon temperature and, consequently, enhanced phonon occupation. (iii) When *e – pht* coupling is large, 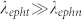, there is no longer efficient transfer of heat to the phonons. However, the D-LUMO occupation continues to rise, due to energy pumping from the photons to the D-LUMO.

An important measure of the operational efficiency of the molecular PV cell is the efficiency at maximal power, *η*_mx_, defined as the ratio between the cell's maximal output power *P*_out_ and the corresponding input power *P*_in_ supplied by the photons^16^,^17^. The output power is given by *P*_out_ = *J* × *V*, where *J* is the particle current through the system, and the input power is calculated in a similar way^4^ from 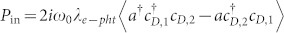, which is related to the photon-induced part of the particle current.

The inset of Fig. 2(b) shows a typical *J* − *V* characteristics (blue) and the bias voltage *V* dependence of the output power (purple). As seen in the figure, there exists a bias voltage *V*_mx_ which gives the maximum efficiency. In Fig. 2(b), the efficiency at *V*_mx_, i.e., the efficiency at maximum power, is plotted as a function of the *e – pht* coupling (we use the same parameters as in Fig. 2(a)). For very small *e* – *pht* coupling (*λ_epht_* < 0.002 eV), the efficiency is very small, and grows linearly with *λ_epht_*. In this regime, the time it takes for an electron to absorb a photon is larger than the time the photons spent in the cell (defined by *γ_pht_n_B_* (*T_s_*) which translates to ~0.002 eV), and so the photon absorption is very small leading to poor efficiency. This regime is followed by a plateau regime, where any energy transferred from the photons to the electrons is quickly dissipated by phonons and is not converted into electrical power. There is thus little change in the efficiency, as long as the rate of photon absorption is smaller than the electron-photon-relaxation time. Only when the *e – pht* coupling reaches the *e – phn* coupling *λ_ephn_* = 0.1 eV the efficiency begins to increase: in this regime, energy is transferred to the electrons by the photons faster than can be dissipated by the phonons, and as a result an increasing amount of this energy is transferred into electronic power, resulting in a rise of efficiency.

It is important to note that the results described in Fig. 2, especially in the region where the electron-phonon and electron-photon couplings are of the same order, *cannot be obtained by assuming equilibrium distributions for the phonons and photons*, and this situation is described here for the first time. Since the strength of the electron-photon and electron-phonon interactions of future realistic devices are unknown, a situation where they are of similar magnitude may occur, in which case the system dynamics cannot be described as close to equilibrium, and the full non-equilibrium dynamics need to be taken into account.

**Figure 3:**
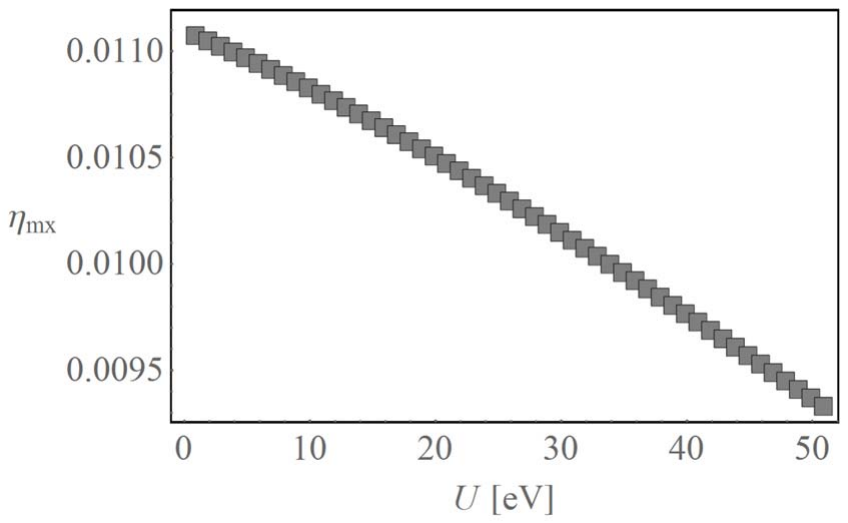
Efficiency at maximum power *η*_mx_ as a function of the exciton Coulomb energy *U*.

To further demonstrate the power of this method, we next discuss the effect of Coulomb interactions on the efficiency. In the Hamiltonian of Eq. (1), the A-LUMO energy, 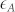, already includes the Coulomb repulsion energy on the acceptor^4^. In excitonic systems, the Coulomb interaction is typically considered through the “exciton binding energy”, defined by the Coulomb interaction term 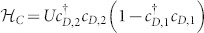 between an electron at the D-LUMO and a hole in the D-HOMO. While in methods such as non-equilibrium Green's function adding Coulomb interaction requires substantial effort, the present method does not require either additional technical complexity or additional computational power to account for any Coulomb interaction effects. In Fig. 3 we show the efficiency at maximum power *η*_mx_ as a function of the exciton Coulomb energy *U* (which can be estimated from, e.g. density-functional calculations). We set *λ_epht_* = 0.1 eV and *λ_ephn_* = 0.2 eV. We find an almost linear decrease in the efficiency, with a reduction of ~15% for *U* = 0.2 eV.

## Results: the role of decoherence

The next question we wish to address is the extent to which the quantum nature of the system affects the PV conversion efficiency, a question which is beyond the reach of the formalism presented in Refs. 4, 5. The formalism we present here allows us to access, in addition to fully quantum-coherent processes described above, also incoherent processes. The most important incoherent processes are electron transfer from the D-LUMO to the A-LUMO, described classically in Ref. 4. These are addressed here by adding an additional pair of 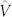
–operators that accounts for incoherent transitions, namely 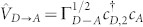, 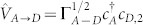, where 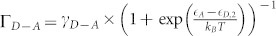 and vice versa. Thus, the pair of parameters *t_D–A_* and *γ_D–A_* describe the strength of the coherent and incoherent donor-acceptor electron transfer processes, respectively.

In what was shown in Fig. 2, the D- and A- LUMO levels were connected by quantum-mechanical bonding. In contrast, Ref. 4 accounted for the electronic transfer between the D- and A- LUMO levels by an incoherent (or classical) transfer process. It is thus of interest to interpolate between the fully quantum case (*t_D–A_* ≠ 0, *γ_D–A_* = 0), through the mixed quantum-classical case (*t_D–A_* ≠ 0, *γ_D–A_* ≠ 0), to the fully classical case (*t_D–A_* = 0, *γ_D–A_* ≠ 0).

**Figure 4:**
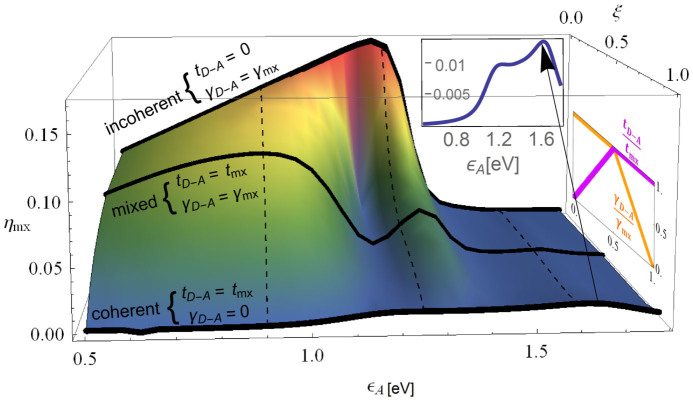
Efficiency at maximum power *η*_mx_ as a function of the position of the A-LUMO 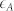 and the parameter *ξ* which describes the interpolation from coherent to incoherent electron D-A processes (see text). Solid lines mark the coherent (*t_D–A_* = *t*_mx_, *γ_D–A_* = 0), mixed (*t_D–A_* = *t*_mx_, *γ_D–A_* = *γ*_mx_) and incoherent (*t_D–A_* = 0, *γ_D–A_ = γ*_mx_) donor-acceptor electron transfer processes. Dashed lines are guides to the eye, showing the position of the maximal efficiency for the different cases. Right inset: parameterization of *t_D–A_* and *γ_D–A_* with *ξ*. Back inset: Efficiency as a function of the position of the A-LUMO 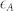 at *ξ* = 0 (fully coherent system).

To do so, we define a variable *ξ* such that 0 ≤ *ξ* ≤ 1, and define *t*_mx_ = 0.05 eV and *γ*_mx_ = 10^12^ s^−1^ (as in Ref. 4). We now parameterize *t_D–A_* and *γ_D–A_* with *ξ*, *t_D–A_* = *t*_mx_ (1 − 2(*ξ* − 0.5)Θ(*ξ* − 0.5)), *γ_D–A_* = *γ*_mx_ (1 − 2(0.5 − *ξ*)Θ(0.5 − *ξ*)) (Θ(*ξ*) is the Heaviside unit step-function). This parameterization is shown on the right inset of Fig. 4, and is constructed such that for *ξ* = 0 the system is fully coherent, for *ξ* = 0.5 the system is mixed (both quantum and classical processes), and for *ξ* = 1 the system is fully incoherent, so the range 0 < *ξ* < 1 interpolates between all three cases.

In Fig. 4 we plot the efficiency at maximum power *η*_mx_ as a function of the position of the A-LUMO, 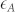 and the parameter *ξ*. We set *γ_D–A_* = 10^12^ s^−1^ as in Ref. 4. We find that the quantum coherence or classical decoherence (parameterized by *ξ*) has a profound effect on the efficiency of the molecular PV-cell in two important aspects.

First, the optimal position of the A-LUMO energy differs according to the nature of the transition under consideration: quantum (coherent), both quantum and classical, or classical D-A transitions (solid lines in Fig. 4). For the last case (*t_D–A_* = 0, *γ_D–A_* = 10^12^ s^−1^), we find that 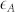 is optimal at 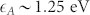, verifying the result of Ref. 4. When quantum correlations are added (*t_D–A_* = 0.05 eV, *γ_D–A_* = 10^12^ s^−1^), two peaks emerge at 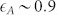, 1.3 eV, and a lower peak emerges at 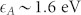. For a system with only quantum transitions (*t_D–A_* = 0.05 eV, *γ_D–A_* = 0, enlarged in the back inset in Fig. 4), the lower peak vanishes, and the optimal LUMO positions are at 1.2 eV and 1.6 eV. Thus, in the design of optimal molecular PV cells, it is important to take into account the quantum nature.

Second, as can be clearly seen in Fig. 4, the addition of classical D-A transitions *increases* the efficiency substantially by more than an order of magnitude. This finding is surprising, since one would expect that incoherent (and dissipative) transitions would lead to a decrease in the efficiency. To understand the origin of this effect, we performed time-dependent calculations (not shown) for a system composed of D-LUMO, acceptor, and the coupling with right electrode, and found that for the coherent case, an electron that is excited to the D-LUMO coherently oscillates between the D- and A-LUMO, while for the incoherent case the electron decays from the D- to the A-LUMO exponentially (and its return rate is exponentially small). This implies that in the coherent case the electron spends much more time in the D-LUMO than in the incoherent case, before transferring to the right electrode. Since the electron can decay back to the D-HOMO (emitting a phonon) only directly from the D-LUMO, the longer it spends on the D-LUMO, the higher the probability for non-radiative decay back to the D-HOMO, leading to a decrease in efficiency. This phenomena is similar to dephasing-assisted transport conjectured to occur in biological systems^18^,^19^,^20^,^21^, but here is the first time it is discussed and demonstrated in the context of molecular PV cells. Since in realistic single-molecule junctions both coherent and incoherent effects may be important, they must be included in a theoretical description of the system.

To illustrate this connection between dynamics and efficiency, we address the relaxation dynamics of the donor-acceptor system. Considering only the D-A LUMOs and the right electrode (without the photons and phonons), we construct the Lindbladian 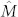-matrix of Eq. (2) (at zero bias), by constructing a vector form for the Lindblad equation of Eq. (2), 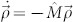. The 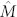-matrix has a zero eigenvalue, which defines the steady state. The (real part of the) rest of the eigenvalues define the relaxation rates towards the steady state. The minimal rate Γ_min_ (i.e., eigenvalues of 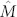 with smallest real part which, we numerically check, is non-zero) represents the longest relaxation time for the system to reach the steady state from any general state (see supplementary material).

**Figure 5:**
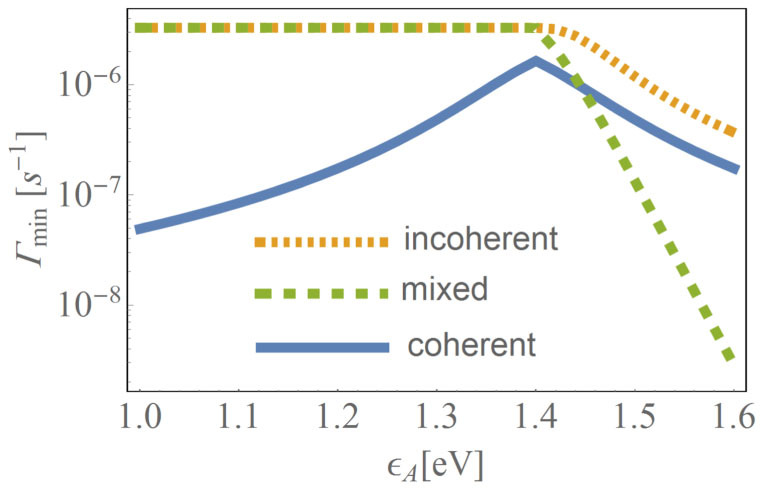
Minimal decay rate Γ_min_ (on a log scale) as a function of the position of the acceptor LUMO, 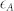, for the three cases of fully coherent (*t_D–A_* = *t*_mx_, *γ_D–A_* = 0), mixed (*t_D–A_* = *t*_mx_, *γ_D–A_* = *γ*_mx_) and incoherent (*t_D–A_* = 0, *γ_D–A_* = *γ*_mx_) donor-acceptor electron transfer processes.

In Fig. 5, we plot the decay rate Γ_min_ of the model which only contains D-A LUMOs and the right electrode (without the photons and phonons) as a function of the position of the A-LUMO 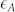 for the three cases of fully coherent (*t_D–A_* = *t*_mx_, *γ_D–A_* = 0), mixed (*t_D–A_* = *t*_mx_, *γ_D–A_* = *γ*_mx_) and incoherent (*t_D–A_* = 0, *γ_D–A_* = *γ*_mx_) donor-acceptor electron transfer processes. As can be seen, the decay rate is much smaller for the fully coherent case, indicating a longer relaxation time. This is in line with the observation above that slower relaxation dynamics lead to lower efficiency. In addition, we point out that the relaxation times of the D-A LUMOs and the right electrode (which are numerically much easier to calculate than the efficiency of the full system including D-HOMO, photons, phonons, and related Lindblad dissipators) serve as an indicator for the efficiency, even though they do not capture the fine details required for an optimal design of the system (see Fig. 4(a) and (b)).

**Figure 6:**
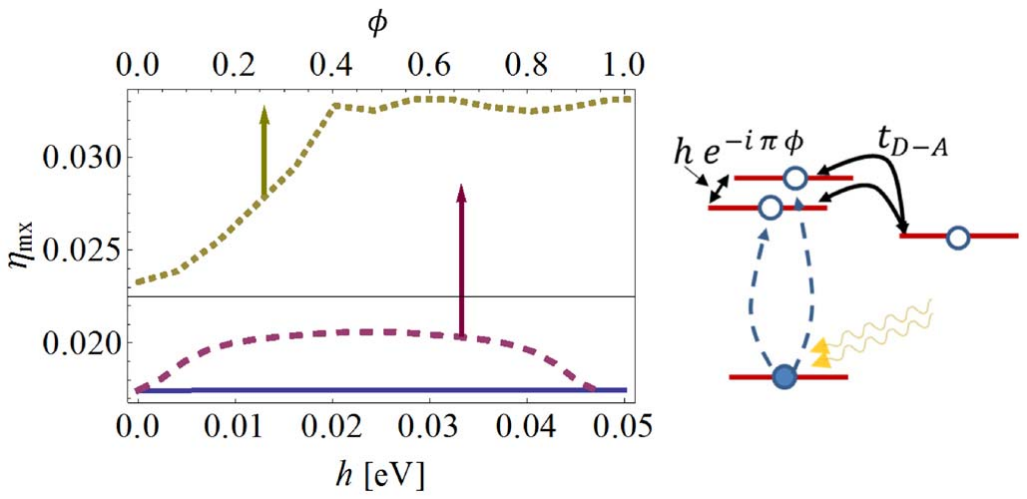
Right: Schematic illustration of the molecular PV cell, with two donor LUMO levels. Left: Efficiency at maximum power *η*_mx_ as a function of hopping amplitude *h* between the donor LUMO levels and the acceptor LUMO (solid line), and hopping matrix element phase *φ* (dashed line), indicating the effect of quantum coherence on the efficiency for fully coherent D-A transfer (*γ_D–A_* = 0). The dotted line is the same for a mixed coherent-incoherent transfer (*γ_D–A_* ≠ 0).

To further examine possible effects of coherence on the efficiency of the molecular PV cell, we study a system where the donor has two degenerate D-LUMO levels which have been introduced experimentally^22^,^23^. Here, we study a simplified system (schematically depicted on the right side of Fig. 6), in which the photons and the phonons excite electrons with equal amplitudes from the D-HOMO to the two D-LUMO levels. Each of the levels is coupled to the A-LUMO with the same hopping amplitude *t_D–A_*, and they are coupled to each other with a complex hopping amplitude *he*^−*iπφ*^. In Fig. 6, the efficiency at maximum power *η*_mx_ is plotted as a function of the inter-LUMO coupling *h* (solid line) and the phase *φ* (dashed line) for *γ_D–A_* = 0, i.e., no incoherent D-A transfer (*λ_ephn_* = *λ_epht_* = 0.1 eV). We find that while *h* has little effect on the efficiency, the phase *φ* has a significant effect (increasing the efficiency by up to ~15%). Surprisingly, we also find that this quantum interference effect persists even when incoherent D-A transfer is included (*γ_D–A_* = 10^12^ s^−1^ as in Fig. 4), and an substantial increase of *η*_mx_ ~ 30% is observed by varying *φ* (dotted line).

## Conclusions

In Summary, we have proposed a novel formalism to study non-equilibrium quantum transport in molecular junctions, and applied it to investigate a minimal model of PV energy conversion in ideal, single-molecule PV cells. The results shown above indicate that quantum coherence effects are important in determining the non-equilibrium energy conversion performance of molecular PV cells. The formalism presented here sets the stage for a fully coherent quantum mechanical calculation of energy conversion in more realistic models for molecular PV cells and can be directly linked to quantum chemistry methods (such as density-functional theory). The progress in the experimental ability to measure photo-conductivity and PV conversion in single-molecule junctions^11^,^12^,^13^,^14^,^15^ allows one to envision real PV devices composed of a single molecular junction or a molecular monolayer, making our theoretical model experimentally relevant. Furthermore, our method can include both coherent and incoherent effects, making it a useful tool in the study of other energy conversion processes such as photosynthesis, where both classical and quantum processes take place^18^,^19^,^20^,^21^,^24^,^25^,^26^,^27^,^28^,^29^,^30^,^31^,^32^,^33^,^34^,^35^,^36^,^37^,^38^, or other chemical and photo-chemical processes^39^,^40^.

## Methods

The full Hamiltonian of the molecular PV cell, including the molecular orbitals, the photons and the phonons, may be written as 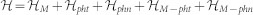 where 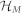 is the Hamiltonian for the molecular complex, 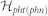 is the photon (phonon) Hamiltonian, and 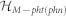 describes the electron-photon (phonon) interaction (we set 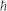 = 1 hereafter),
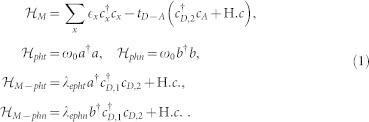
Here 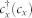 creates (annihilates) an electron in the D-HOMO (*x* = *D*, 1), D-LUMO state (*x* = *D*, 2) or A-LUMO state (*x* = *A*), with the corresponding level energies 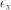, *a*^†^(*a*) creates (annihilates) a photon with energy 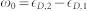, and *b*^†^(*b*) creates (annihilates) a phonon with the same energy. In principle one should consider many photon (and phonon) modes, however the strongest effect on the dynamics comes from the resonant photons (with energy same as the HOMO-LUMO gap). The electron-photon Hamiltonian 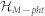 describes (within the rotating wave approximation) the process (and its reverse process) in which an electron in the D-LUMO state relaxes to the D-HOMO state and emits a photon, with the electron-photon (*e – pht*) coupling *λ_epht_*. The electron-phonon Hamiltonian 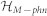 is similar to 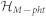, but with phonons instead of photons. While spins might play a role in real systems (for example by introducing selection rules for allowed transitions), we chose to introduce a simplified (toy) model in which spins play no role in the transport processes^4^,^7^.

To study the dynamics of the system, we use the Lindblad equation to model the system and the environments^8^,^9^,^10^,^41^,^42^.
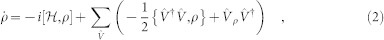
where [·, ·] is the commutator and {·, ·} is the anti-commutator.

The essence of the Lindblad approach is that instead of describing the environment by encoding it into a self-energy (as is done in the non-equilibrium Green's function approach^42^) the environment is characterized by its action on the system. This action is mapped onto so-called Lindblad 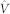-operators, which describe incoherent transitions of the system elements due to the presence of an environment. The Lindblad equation was recently employed to address various aspects of electron transport^43^,^44^,^45^,^46^,^47^,^48^,^49^, yet in these studies the interaction with an environment was limited to electrons only, and the non-equilibrium dynamics of other constituents (i.e. phonons or photons) was not considered.

We assume that the left electrode is coupled only to the D-HOMO and that the right electrode is coupled only to the A-LUMO^4^, as in Fig. 1. The corresponding 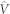-operators are then^44^,^48^,^49^:
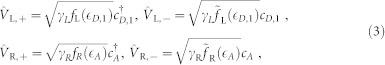
where *γ*_L,R_ are electron transfer rates to the left and right electrodes, *T* is the ambient temperature (we take *T* = 300 K), *μ*_L_ = 0 is the left-electrode chemical potential, *μ*_R_ = *V* is the right electrode chemical potential, *V* is the bias voltage, 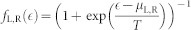 are the Fermi-Dirac distributions of the left and right electrodes, and 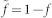. Following Refs. 4, 5 we set *γ*_L_ = *γ*_R_ = 10^10^ s^−1^ (which corresponds to an energy scale of ~4 × 10^−^5 eV, much smaller than the other electronic energy scales, validating the use of the Lindblad equations).

For the bosons (photons and phonons), similar 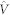-operators that relate to the Bose-Einstein statistics of the boson baths are constructed,
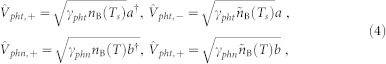
where *γ_pht_*, *γ_phn_* are photon and phonon relaxation rates (set to *γ_pht_* = *γ_phn_* = 10^12^ s^−1^), *T_s_* ~ 5700 K is the solar temperature, 
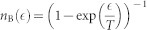
 is the Bose-Einstein distribution, and 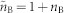.

Once the 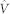-operators are set, the dynamics are determined by the propagation of the density matrix via the Lindblad equation. We numerically study the steady-state density matrix *ρ*_SS_ with truncated bosonic space containing *n* excitations and find that all the physical properties converge at *n* = 6. Therefore, we demonstrate our results with *n* = 6 (for comparison, in Ref. 4 the photons and phonons were treated in a non-self-consistent mean-field approximation). The expression for the current is obtained from the formal continuity equation 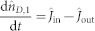, where 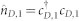. The resulting expression for the current is 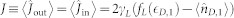, where 〈·〉 represents the steady state average. Equivalently, the current 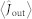 can be written as the sum of photon-induced and phonon-induced current, 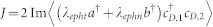.

## Author Contributions

Y.D. conceived the research and drafted the paper. S.A. performed the numerical and analytical work together with B.Z. All authors discussed the results and contributed to the final version of the manuscript.

## Supplementary Material

Supplementary Information Supplementary Material

## References

[b1] Gratzel M. Solar energy conversion by dye-sensitized photovoltaic cells. Inorganic Chemistry 44, 6841–6851 (2005). 16180840 10.1021/ic0508371

[b2] Deibel C. & Dyakonov V. Polymer-fullerene bulk heterojunction solar cells. Reports on Progress in Physics 73, 096401 (2010).

[b3] Nicholson P. G. & Castro F. A. Organic photovoltaics: principles and techniques for nanometre scale characterization. Nanotechnology 21, 492001 (2010). 21071826 10.1088/0957-4484/21/49/492001

[b4] Einax M., Dierl M. & Nitzan A. Heterojunction organic photovoltaic cells as molecular heat engines: A simple model for the performance analysis. The Journal of Physical Chemistry C 115, 21396–21401 (2011).

[b5] Einax M., Dierl M., Schiff P. R. & Nitzan A. Multiple state representation scheme for organic bulk heterojunction solar cells: A novel analysis perspective. EPL (Europhysics Letters) 104, 40002 (2013).

[b6] Smirnov A. Y., Mourokh L. G., Ghosh P. K. & Nori F. High-efficiency energy conversion in a molecular triad connected to conducting leads. The Journal of Physical Chemistry C 113, 21218–21224 (2009).

[b7] Rutten B., Esposito M. & Cleuren B. Reaching optimal efficiencies using nanosized photoelectric devices. Phys. Rev. B 80, 235122 (2009).

[b8] van Kampen N. G. Stochastic Processes in Physics and Chemistry (North Holland; 3 edition, 2007).

[b9] Lindblad G. On the enerators of quantum dynamical semigroups. Communications in Mathematical Physics 48, 119–130 (1976).

[b10] Gorini V., Kossakowski A. & Sudarshan E. C. G. Completely positive dynamical semigroups of n-level systems. Journal of Mathematical Physics 17, 821–825 (1976).

[b11] Battacharyya S. *et al.* Optical modulation of molecular conductance. Nano Letters 11, 2709–2714 (2011). 21657259 10.1021/nl200977c

[b12] Fereiro J. A., McCreery R. L. & Bergren A. J. Direct optical determination of interfacial transport barriers in molecular tunnel junctions. Journal of the American Chemical Society 135, 9584–9587 (2013). 23782345 10.1021/ja403123a

[b13] Furmansky Y. *et al.* Porphyrins as ito photosensitizers: substituents control photo-induced electron transfer direction. J. Mater. Chem. 22, 20334–20341 (2012).

[b14] Aradhya S. V. & Venkataraman L. Single-molecule junctions beyond electronic transport. Nat Nano 8, 399–410 (2013). 10.1038/nnano.2013.91 23736215

[b15] Gerster D. *et al.* Photocurrent of a single photosynthetic protein. Nat Nano 7, 673–676 (2012). 10.1038/nnano.2012.165 23023644

[b16] Esposito M., Lindenberg K. & Van den Broeck C. Universality of efficiency at maximum power. Phys. Rev. Lett. 102, 130602 (2009). 19392340 10.1103/PhysRevLett.102.130602

[b17] Van den Broeck C. Thermodynamic efficiency at maximum power. Phys. Rev. Lett. 95, 190602 (2005). 16383969 10.1103/PhysRevLett.95.190602

[b18] Caruso F., Chin A. W., Datta A., Huelga S. F. & Plenio M. B. Highly efficient energy excitation transfer in light-harvesting complexes: The fundamental role of noise-assisted transport. The Journal of Chemical Physics 131, 105106 (2009).

[b19] Mohseni M., Rebentrost P., Lloyd S. & Aspuru-Guzik A. Environment-assisted quantum walks in photosynthetic energy transfer. The Journal of chemical physics 129, 174106 (2008). 19045332 10.1063/1.3002335

[b20] Rebentrost P., Mohseni M., Kassal I., Lloyd S. & Aspuru-Guzik A. Environment-assisted quantum transport. New Journal of Physics 11, 033003 (2009).

[b21] Plenio M. B. & Huelga S. F. Dephasing-assisted transport: quantum networks and biomolecules. New Journal of Physics 10, 113019 (2008).

[b22] Rizzi A. C. *et al.* Entropic changes control the charge separation process in triads mimicking photosynthetic charge separation. The Journal of Physical Chemistry A 112, 4215–4223 (2008). 18402483 10.1021/jp712008b

[b23] Terazono Y. *et al.* Photoinduced electron transfer in a hexaphenylbenzene-based self-assembled porphyrin-fullerene triad. Photochemistry and Photobiology 83, 464–469 (2007). 17576351 10.1562/2006-12-05-RC-1098

[b24] Abramavicius D., Voronine D. & Mukamel S. Unravelling coherent dynamics and energy dissipation in photosynthetic complexes by 2d spectroscopy. Biophys. J. 94, 3613 (2008). 18192357 10.1529/biophysj.107.123455 PMC2292377

[b25] Ai B.-Q. & Zhu S.-L. Complex quantum network model of energy transfer in photosynthetic complexes. Phys. Rev. E 86, 061917 (2012). 10.1103/PhysRevE.86.061917 23367985

[b26] Cheng Y.-C. & Fleming G. R. Dynamics of light harvesting in photosynthesis. Annual Review of Physical Chemistry 60, 241–262 (2009). 10.1146/annurev.physchem.040808.090259 18999996

[b27] Chin A., Huelga S. & Plenio M. Coherence and decoherence in biological systems: principles of noise-assisted transport and the origin of long-lived coherences. Philosophical Transactions of the Royal Society A: Mathematical, Physical and Engineering Sciences 370, 3638–3657 (2012). 10.1098/rsta.2011.0224 22753818

[b28] Collini E. *et al.* Coherently wired light-harvesting in photosynthetic marine algae at ambient temperature. Nature 463, 644–647 (2010). 20130647 10.1038/nature08811

[b29] Engel G. S. *et al.* Evidence for wavelike energy transfer through quantum coherence in photosynthetic systems. Nature 446, 782–786 (2007). 17429397 10.1038/nature05678

[b30] Hoyer S., Sarovar M. & Whaley K. B. Limits of quantum speedup in photosynthetic light harvesting. New Journal of Physics 12, 065041 (2010).

[b31] Ishizaki A. & Fleming G. R. Quantum coherence in photosynthetic light harvesting. Annu. Rev. Condens. Matter Phys. 3, 333–361 (2012). 10.1098/rsta.2011.0207 22753820

[b32] Lambert N. *et al.* Quantum biology. Nature Physics 9, 10–18 (2013).

[b33] Lee H., Cheng Y. & Fleming G. Coherence dynamics in photosynthesis: protein protection of excitonic coherence. Science 316, 1462 (2007). 17556580 10.1126/science.1142188

[b34] Olaya-Castro A., Lee C. F., Olsen F. F. & Johnson N. F. Efficiency of energy transfer in a light-harvesting system under quantum coherence. Physical Review B 78, 085115 (2008).

[b35] Read E., Schlau-Cohen G., Engel G., Wen J. & Blankenship R. Visualization of excitonic structure in the fenna-matthews-olson photosynthetic complex by polarization-dependent two-dimensional electronic spectroscopy. Biophys. J. 95, 847 (2008). 18375502 10.1529/biophysj.107.128199 PMC2440448

[b36] Scholes G., Curutchet C., Mennucci B., Cammi R. & Tomasi J. How solvent controls electronic energy transfer and light harvesting. J. Phys. Chem. B 111, 6978 (2007). 17550286 10.1021/jp072540p

[b37] Calhoun T. R. *et al.* Quantum coherence enabled determination of the energy landscape in light-harvesting complex ii. The Journal of Physical Chemistry B 113, 16291–16295 (2009). 20014871 10.1021/jp908300c

[b38] Caruso F., Chin A. W., Datta A., Huelga S. F. & Plenio M. B. Entanglement and entangling power of the dynamics in light-harvesting complexes. Phys. Rev. A 81, 062346 (2010).

[b39] Miller W. H. Perspective: Quantum or classical coherence? The Journal of Chemical Physics 136, 210901 (2012). 22697519 10.1063/1.4727849

[b40] Xie X. *et al.* Attosecond-recollision-controlled selective fragmentation of polyatomic molecules. Phys. Rev. Lett. 109, 243001 (2012). 23368312 10.1103/PhysRevLett.109.243001

[b41] Breuer H.-P. & Petruccione F. The Theory of Open Quantum Systems (Oxford University Press, USA, 2002).

[b42] Di Ventra M. Electrical Transport in Nanoscale Systems (Cambridge University Press, 2008).

[b43] Ajisaka S. & Barra F. Nonequilibrium mesoscopic fermi-reservoir distribution and particle current through a coherent quantum system. Physical Review B 87, 195114 (2013).

[b44] Ajisaka S., Barra F., Mejia-Monasterio C. & Prosen T. Nonequlibrium particle and energy currents in quantum chains connected to mesoscopic fermi reservoirs. Phys. Rev. B 86, 125111 (2012).

[b45] Dzhioev A. A. & Kosov D. Stability analysis of multiple nonequilibrium fixed points in self-consistent electron transport calculations. The Journal of chemical physics 135, 174111 (2011). 22070296 10.1063/1.3658736

[b46] Dzhioev A. A. & Kosov D. Nonequilibrium configuration interaction method for transport in correlated quantum systems. Journal of Physics A: Mathematical and Theoretical 47, 095002 (2014).

[b47] Dzhioev A. A. & Kosov D. Second-order post-hartree–fock perturbation theory for the electron current. The Journal of chemical physics 134, 154107 (2011). 21513375 10.1063/1.3581098

[b48] Dzhioev A. A. & Kosov D. S. Super-fermion representation of quantum kinetic equations for the electron transport problem. The Journal of Chemical Physics 134, 044121 (2011). 21280701 10.1063/1.3548065

[b49] Harbola U., Esposito M. & Mukamel S. Quantum master equation for electron transport through quantum dots and single molecules. Physical Review B 74, 235309 (2006).

