# Supplementary material for: The Molecular Photo-Cell: Quantum Transport and Energy Conversion at Strong Non-Equilibrium
Source: Sci Rep. 2015 Feb 9;5:8312. doi: 10.1038/srep08312 (PMC4321170; doi:10.1038/srep08312)
Supplement: Supplementary Information — Supplementary Material [file srep08312-s1.pdf]

# The Molecular Photo-Cell: Quantum Transport and Energy Conversion at Strong Non-Equilibrium

## Supplementary Material

Shigeru Ajisaka
*Department of Chemistry, Ben-Gurion University of the Negev, Beer-Sheva 84105, Israel*

Bojan Žunkovič
*Departamento de Física, Facultad de Ciencias Físicas y Matemáticas,*
*Universidad de Chile, Casilla 487-3, Santiago Chile*

Yonatan Dubi *
*Department of Chemistry and Ilse-Katz Institute for Nanoscale Science and Technology,*
*Ben-Gurion University of the Negev, Beer-Sheva 84105, Israel*
(Dated: November 29, 2014)

## SUPPLEMENTAL

In this supplementary material, we shall review the background and the calculation of the main part. We shall also discuss the definition of the efficiency and the conservation law.

## GENERAL THEORY

In the main part, we have used the Lindblad equation to describe a coupling between the system and environments, namely, to open the system. Mathematically, the Lindblad equation is proven to be the completely positive trace preserving (CPTP) Markovian dynamics. On the contrary, any Markovian CPTP can be written with respect to the Lindblad equation [1, 2]. Therefore, any Markovian time evolution should be described by the Lindblad form.

Following [3], we review the connection to the open system. Let us consider a system S coupled to reservoirs $H_{\mathrm{B}}$ with interaction $V$. The integration of the interaction picture Von Neumann equation of the total system reads

$$\rho^{\mathrm{I}}_{\mathrm{tot}}(t) = \rho_{\mathrm{tot}}(0) - i\int_0^t [V_{\mathrm{I}}(s), \rho^{\mathrm{I}}_{\mathrm{tot}}(s)]ds \ , \tag{1}$$

where $\rho^{\mathrm{I}}_{\mathrm{tot}}(t)$ denotes an interaction picture density operator, and $V_{\mathrm{I}}(t)$ is an interaction picture of $V$. Hereafter, we shall assume that the density matrix is well approximated by a product state, i.e., $\rho_{\mathrm{tot}}(t) \approx \rho_{\mathrm{I}}(t) \otimes \rho_{\mathrm{B}}$. Substituting Eq. 1 into interaction picture Von Neuman equation, and tracing out the reservoirs, we get

$$\dot{\rho}_{\mathrm{I}}(t) = -\int_0^t \mathrm{tr}_{\mathrm{B}}\big[V_{\mathrm{I}}(t), [V_{\mathrm{I}}(s), \rho_{\mathrm{I}}(s)\otimes\rho_{\mathrm{B}}]\big]ds \ , \tag{2}$$

where $tr_B(\cdot)$ denotes the partial trace over the reservoirs. Replacing $\rho_{\mathrm{I}}(s)$ on the rhs of Eq. (2) by $\rho_{\mathrm{I}}(t)$ and changing an integration valuable give the Redfield equation,

$$\dot{\rho}_{\mathrm{I}}(t) = -\int_0^t \mathrm{tr}_{\mathrm{B}}\big[V_{\mathrm{I}}(t), [V_{\mathrm{I}}(t-s), \rho_{\mathrm{I}}(t)\otimes\rho_{\mathrm{B}}]\big]ds \ . \tag{3}$$

We note that the Redfield equation does not preserve the positivity of the density operator in general.

Extension of the integration regime from $[0, t]$ to $[0, \infty]$ gives a Markovian equation. We further assume $[\rho_{\mathrm{B}}, H_{\mathrm{B}}] = 0$, then, the Markovian master equation is cast into the form

$$\begin{aligned}
\dot{\rho}_{\mathrm{I}}(t) &= \sum_{\omega,\omega'}\sum_{\alpha,\beta} e^{i(\omega'-\omega)t}\Gamma_{\alpha,\beta}(\omega)(A_\beta(\omega)\rho_{\mathrm{I}}(t)A^\dagger_\alpha(\omega') - A^\dagger_\alpha(\omega')A_\beta(\omega)\rho_{\mathrm{I}}(t)) + (\mathrm{H.}c.) \ , \\
V &= \sum_\alpha A_\alpha \otimes B_\alpha, \qquad A_\alpha(\omega) = \sum_{\epsilon'-\epsilon=\omega} \Pi(\epsilon)A_\alpha\Pi(\epsilon') \ , \\
\Gamma_{\alpha,\beta}(\omega) &= \int_0^\infty ds e^{i\omega s}\mathrm{tr}_{\mathrm{B}}\left(B^\dagger_\alpha(s)B_\beta(0)\rho_{\mathrm{B}}\right) \ ,
\end{aligned} \tag{4}$$

where $\Pi(\epsilon)$ denotes the projection onto the eigenspace of $H_{\mathrm{S}}$ with the eigenvalue $\epsilon$. By neglecting terms with $\omega \neq \omega'$ in Eq. (4) (the rotating wave approximation), we have

$$\dot{\rho}_{\mathrm{I}}(t) = -i\left[\sum_{\omega,\alpha,\beta} \mathrm{Im}\left(\Gamma_{\alpha,\beta}(\omega)\right) A^\dagger_\alpha(\omega)A_\beta(\omega), \rho_{\mathrm{I}}(t)\right] + \hat{\mathcal{D}}\left(\rho_{\mathrm{I}}(t)\right) \ , \tag{5}$$

$$\hat{\mathcal{D}}\left(\rho_{\mathrm{I}}(t)\right) \equiv \sum_{\omega,\alpha,\beta} \mathrm{Re}\left(\Gamma_{\alpha,\beta}(\omega)\right)\Big(2A_\beta(\omega)\rho_{\mathrm{I}}(t)A^\dagger_\alpha(\omega) - \big\{A^\dagger_\alpha(\omega)A_\beta(\omega), \rho_{\mathrm{I}}(t)\big\}\Big) \ . \tag{6}$$

This is the Lindblad equation in the interaction picture. We note that the matrix $\mathrm{Re}\left(\Gamma_{\alpha,\beta}\right)$ should be positive [3], which is consistent with the mathematical derivation.

As shown above, one should choose the Lindblad operator (referred to as $\hat{V}$ operator in the main part) using the eigen energy basis in principle. However, in practical use, the local Lindblad operator is easier to construct. Namely, we choose the Lindblad operator so as to thermalize local elements if they are not coupled to the other constituents. Typically this choice of the Lindblad operator forces the system to a nearly local equilibrium state (local detailed balance) [4]. Indeed, Fig. 2(a) in the main text shows the local thermalization of photon and phonon in a weak coupling regime.

## STEADY STATE CALCULATION OF OUR MODEL

In this section, we emonstrate how we calculate the steady state of our model. Let us consider the density operator $\rho$ which obeys the Lindblad equation. We focus on the density operator acting on a finite dimensional Hilbert space $\mathcal{H}$ with dimension $N$. In this case, the density operator is an $N \times N$ positive matrix with trace one. We vectorize the density operator $\rho$ with $N^2$ dimensional vector $\vec{\rho}_{i+jN} = \rho_{i,j}$. Then, the left/right multiplication $\hat{L}(A)/\hat{R}(A)$ of the operator $A$ is represented d by Kronecker products of following matrices

$$\hat{L}(A) = A \otimes \mathbf{I}_n,\ \hat{R}(A) = \mathbf{I}_n \otimes A^T \ , \tag{7}$$

where $\mathbf{I}_N$ is an $N$ dimensional identity matrix. For D-LUMO, D-HOMO, A, photon, and phonon with truncated level $n$, the Hilbert space dimension is $N = 2^3 n^2$. The Lindblad equation is equivalent to the following $N^2$ dimensional linear ordinary differential equation with time independent constant.

$$\dot{\vec{\rho}} = -\hat{M}\vec{\rho} \tag{8}$$

We remark that the dimension of the vectorized density operator $\vec{\rho}$ is $N^2$, and that of the Liouvillian map $(-\hat{M})$ is $N^4$. The Lindblad form guarantees that eigen values $\{\lambda_i\}_{i=1}^{N^4}$ of the matrix $\hat{M}$ are non-negative, i.e., $\lambda_i \geq 0$. The trace preserving property implies that at least one zero eigen value. In our calculation, we have checked that there is only one eigen value with zero real part (essentially the zero coming from trace preserving property), therefore, the corresponding eigen vector is a unique steady state of the model. The relaxation time to the steady state is given by $\min_i \mathrm{Re}\ \lambda_i > 0$, where $i$ corresponding to the steady state is excluded in the minimum. By reforming the vector to matrix again, one gets the steady state $\rho_{SS}$ discussed in the main part.

## CONSERVATION LAW AND THE EFFICIENCY

In this section, we shall discuss the definition of the efficiency. First we shall discuss the particle current. Let $\hat{n}_{D,\mathrm{HOMO}} = c^\dagger_{D,1} c_{D,1}$, $\hat{n}_{D,\mathrm{LUMO}} = c^\dagger_{D,2} c_{D,2}$, and $\hat{n}_A = c^\dagger_A c_A$ , then, their time derivatives obey the Heisenberg equation.

$$\frac{\mathrm{d}}{\mathrm{d}t}\hat{n}_{D,\mathrm{HOMO}} = -\hat{J}_{pht} - \hat{J}_{phn} + \hat{\mathcal{D}}_{\mathrm{H}}(\hat{n}_{D,\mathrm{HOMO}}) \tag{9}$$

$$\frac{\mathrm{d}}{\mathrm{d}t}\hat{n}_{D,\mathrm{LUMO}} = \hat{J}_{pht} + \hat{J}_{phn} - \hat{J}_{D-A} \tag{10}$$

$$\frac{\mathrm{d}}{\mathrm{d}t}\hat{n}_A = \hat{J}_{D-A} + \hat{\mathcal{D}}_{\mathrm{H}}(\hat{n}_A) \tag{11}$$

$$\hat{J}_{pht} \equiv i[\hat{n}_{D,\mathrm{HOMO}}, \mathcal{H}_M] = i(a^\dagger c^\dagger_{D,1} c_{D,2} - a c^\dagger_{D,2} c_{D,1}), \tag{12}$$

$$\hat{J}_{phn} \equiv i[\hat{n}_{D,\mathrm{HOMO}}, \mathcal{H}_{phn}] = i(b^\dagger c^\dagger_{D,1} c_{D,2} - b c^\dagger_{D,2} c_{D,1}) \ , \tag{13}$$

$$\hat{J}_{D-A} \equiv i[\hat{n}_{D,\mathrm{LUMO}}, \mathcal{H}_{phn}] = i t_{D-A}(c^\dagger_A c_{D,2} - c^\dagger_{D,2} c_A) \ , \tag{14}$$

where $\hat{\mathcal{D}}_{\mathrm{H}}(\cdot)$ denotes the Heisenberg picture of the Lindblad dissipator. The dissipator comes from the openness, and thus represents the current between system and reservoirs. For instance, the first equation demonstrates that the particle current from the left reservoir to the D-HOMO is given by $\hat{\mathcal{D}}_{\mathrm{H}}(\hat{n}_{D,\mathrm{HOMO}})$, and the particle current from D-HOMO to D-LUMO is a sum of photon related part, $\hat{J}_{pht}$, and phonon related part, $\hat{J}_{phn}$. The current from D-LUMO to D-HOMO is, in general, not equal to that from the left reservoir. They are equal only at steady state since the derivative of $\langle \hat{n}_{D,\mathrm{HOMO}} \rangle$ should be zero. Finally, using the current flowing through the molecule $\hat{J}_{pht} + \hat{J}_{phn}$ and the bias voltage $V$, we obtain a output power

$$\hat{P}_{\mathrm{out}} = (\hat{J}_{pht} + \hat{J}_{phn})V \ . \tag{15}$$

The input power is essentially an energy flow from photon reservoir to the molecule. The time derivative of the photon Hamiltonian follows

$$\frac{\mathrm{d}}{\mathrm{d}t}\mathcal{H}_{pht} = -\omega \hat{J}_{pht} + \hat{\mathcal{D}}_{\mathrm{H}}(\mathcal{H}_{pht}) \ . \tag{16}$$

Similar to the particle conservation law, the first term $\hat{\mathcal{D}}_{\rm H}(\mathcal{H}_{pht})$ represents the energy flow from the photon reservoir to the photon, and the energy flow from the photon to the molecule is given by $\omega\hat{J}_{pht}$, i.e., $\hat{P}_{\rm in} = \omega\hat{J}_{pht}$. Finally, the efficiency is given by

$$\eta \equiv \frac{\langle\hat{P}_{\rm out}\rangle}{\langle\hat{P}_{\rm in}\rangle} = \frac{V\langle\hat{J}_{pht}+\hat{J}_{phn}\rangle}{\omega\langle\hat{J}_{pht}\rangle} \ . \tag{17}$$

We note that $\langle\hat{J}_{phn}\rangle$ typically has a negative sign and the transfer is interrupt by the energy transfer from the molecule to the phonon reservoir through phonon.


[1] Lindblad, G. On the enerators of quantum dynamical semigroups. Communications in Mathematical Physics **48**, 119–130 (1976).

[2] Gorini, V., Kossakowski, A. & Sudarshan, E. C. G. Completely positive dynamical semigroups of nlevel systems. Journal of Mathematical Physics **17**, 821–825 (1976). URL `http://scitation.aip.org/content/aip/journal/jmp/17/5/10.1063/1.522979`.

[3] Breuer, H.-P. & Petruccione, F. The Theory of Open Quantum Systems (Oxford University Press, USA, 2002).

[4] Ajisaka, S., Barra, F. & Žunkovič, B. Nonequilibrium Quantum Phase Transitions in the XY model: comparison of unitary time evolution and reduced density matrix approaches. New Journal of Physics **16**, 033028 (2014).
